# The Role of Lipid and Lipoprotein Metabolism in Non-Alcoholic Fatty Liver Disease

**DOI:** 10.3390/children4060046

**Published:** 2017-06-06

**Authors:** Francesco M. Perla, Maurizia Prelati, Michela Lavorato, Daniele Visicchio, Caterina Anania

**Affiliations:** Policlinico Umberto I Hospital, Sapienza University of Rome, Rome 00161, Italy; maurizia.prelati@libero.it (M.P.); michela.lavorato@gmail.com (M.L.); daniele.visicchio@gmail.com (D.V.); caterina.anania@uniroma1.it (C.A.)

**Keywords:** nonalcoholic fatty liver disease, lipids, lipid metabolism

## Abstract

Due to the epidemic of obesity across the world, nonalcoholic fatty liver disease (NAFLD) has become one of the most prevalent chronic liver disorders in children and adolescents. NAFLD comprises a spectrum of fat-associated liver conditions that can result in end-stage liver disease and the need for liver transplantation. Simple steatosis, or fatty liver, occurs early in NAFLD and may progress to nonalcoholic steatohepatitis, fibrosis and cirrhosis with increased risk of hepatocellular carcinoma. The mechanism of the liver injury in NAFLD is currently thought to be a “multiple-hit process” where the first “hit” is an increase in liver fat, followed by multiple additional factors that trigger the inflammatory activity. At the onset of disease, NAFLD is characterized by hepatic triglyceride accumulation and insulin resistance. Liver fat accumulation is associated with increased lipotoxicity from high levels of free fatty acids, free cholesterol and other lipid metabolites. As a consequence, mitochondrial dysfunction with oxidative stress and production of reactive oxygen species and endoplasmic reticulum stress-associated mechanisms, are activated. The present review focuses on the relationship between intra-cellular lipid accumulation and insulin resistance, as well as on lipid and lipoprotein metabolism in NAFLD.

## 1. Introduction

Nonalcoholic fatty liver disease (NAFLD) comprises a spectrum of fat-associated liver conditions that can result in end-stage liver disease and the need for liver transplantation [1]. Simple steatosis, or fatty liver, occurs early in NAFLD and may progress to nonalcoholic steatohepatitis (NASH), fibrosis and cirrhosis with increased risk of hepatocellular carcinoma [1]. The world-wide epidemic of obesity has led to nonalcoholic fatty liver disease (NAFLD) becoming one of the most prevalent chronic liver disorders in children and adolescents [2,3]. According to a recent systematic review and meta-analysis, the pooled mean prevalence of NAFLD in children from general population studies has been found to be 7.6% (95% confidence intervals (CIs): 5.5% to 10.3%) and 34.2% (95% CIs: 27.8% to 41.2%) in studies based on child obesity clinics [4]. The progression from NAFLD to NASH remains unclear in both children and adults [5]. Approximately 15%–20% of adult patients with NASH will subsequently develop liver fibrosis and cirrhosis, but there are no equivalent long-term follow-up studies in children [6].

The liver is one of the main ectopic sites where lipids accumulate in obese subjects. In particular, ectopic fat accumulation occurs when the energy storage capacity of the adipose tissue is exceeded, leading to increased net lipid flux to non-adipose organs, thereby causing lipotoxicity and insulin resistance [7,8]. As is found in adults, children and adolescents with fatty liver suffer insulin resistance, glucose intolerance, hypertension, and dyslipidemia (high plasma triglyceride and low levels of high density lipoprotein cholesterol) [9,10]. Thus, NAFLD has emerged as the hepatic component of the metabolic syndrome [11], and a strong cardiovascular risk factor even at a very early age [12,13]. Indeed, studies have reported associations between NAFLD and subclinical atherosclerosis and between NAFLD and cardiac function alterations, independently of established risk factors in youth [14,15,16].

The mechanism of the liver injury in NAFLD is currently thought to be a “multiple-hit process” where the first “hit” is represented by an increase in liver fat, followed by multiple additional factors that trigger inflammatory activity [17]. Indeed, at the onset of disease, NAFLD is characterized by hepatic triglyceride accumulation and insulin resistance, which is markedly influenced by hypercaloric diets, sedentary lifestyle, and genetic susceptibility. Liver fat accumulation is associated with increased lipotoxicity from the high levels of free fatty acids (FAs), free cholesterol and other lipid metabolites. As a consequence, mitochondrial dysfunction with oxidative stress and production of reactive oxygen species and endoplasmic reticulum (ER) stress-associated mechanisms are activated [18]. The present review focuses on the relationship between intra-cellular lipid accumulation and insulin resistance, as well as on lipid and lipoprotein metabolism in NAFLD.

## 2. Intra-Cellular Lipid Accumulation and Insulin Resistance

Insulin has important metabolic effects in several organ systems. The term “insulin resistance” is generally utilized to describe impaired insulin-mediated glucose uptake in skeletal muscle. Insulin resistance associated with obesity and NAFLD also involves the liver and adipose tissue. Hepatic insulin resistance is characterized by impaired insulin-mediated suppression of glucose production, whereas adipose tissue insulin resistance is characterized by impaired insulin-mediated suppression of lipolysis.

**Figure 1:**
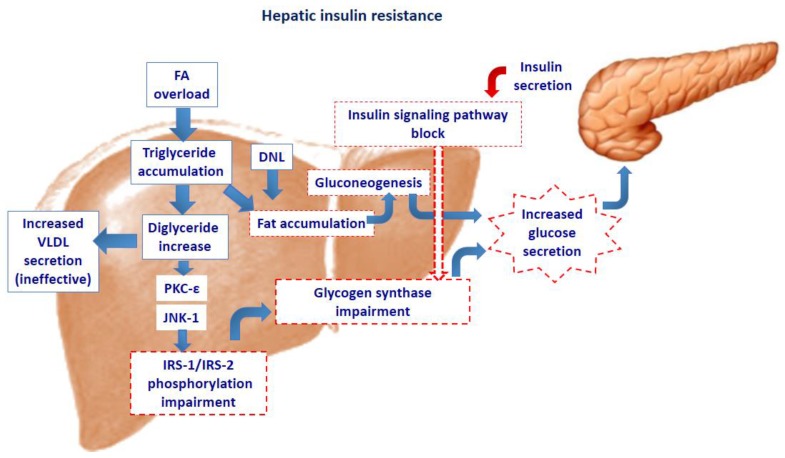
Fatty acid overload in the hepatocyte activates the protein kinase c- epsilon (PKC-ε) and/or c-Jun N-terminal kinase (JNK-1), with subsequent impairment of phosphorylation of the insulin receptor substrate (IRS)-1 and IRS-2. This results in impairment of the insulin signaling pathway. The decreased insulin action on glycogen synthase induces increased glucose secretion. Yet, fat accumulation stimulates neoglucogenesis further increasing the hepatic glucose secretion. The hepatocyte’s attempt to dispose of excessive triglyceride accumulation through increased VLDL secretion, is ineffective, further contributing to accumulation of liver fat. DNL: de novo lipogenesis.

Animal studies have shown that just a few days of high fat diet are sufficient to induce hepatic steatosis and hepatic insulin resistance. In particular, Samuel et al. [19,20] have found that three days of high fat feeding in rats specifically cause hepatic fat accumulation and hepatic insulin resistance without significant peripheral fat accumulation or peripheral insulin resistance. Notably, these changes were not associated with an increase in visceral fat mass or portal vein fatty acid concentrations. Therefore, fat-induced hepatic insulin resistance may be due to activation of the protein kinase C (PKC)-epsilon and/or c-Jun N-terminal kinase-1, highly expressed in liver, which may in turn lead to impaired insulin receptor substrate (IRS)-1 and IRS-2 tyrosine phosphorylation by the insulin receptor kinase (IRTK). The impairment of the insulin signaling pathway then limits the ability of insulin to activate glycogen synthase. Additionally, fat accumulation contributes to increased gluconeogenesis and therefore to total endogenous glucose production [19,20]. These results were confirmed in human studies. In order to evaluate the cellular mechanisms that link hepatic steatosis to insulin resistance, Kumashiro et al. [21] have assessed the role of inflammation, ER stress, and accumulation of hepatocellular lipids in flash-frozen liver biopsies from 37 obese, non-diabetic individuals with NAFLD. They found that hepatic diacylglycerol (DAG) content within cytosolic lipid droplets best accounted for insulin resistance, being responsible for 64% variability in insulin sensitivity. Moreover, the DAG content in lipid droplets was strongly associated with PKC-epsilon activation. These findings were confirmed in another study showing that DAG content was the best predictor of hepatic insulin resistance in obese individuals [22] (Figure 1).

**Figure 2:**
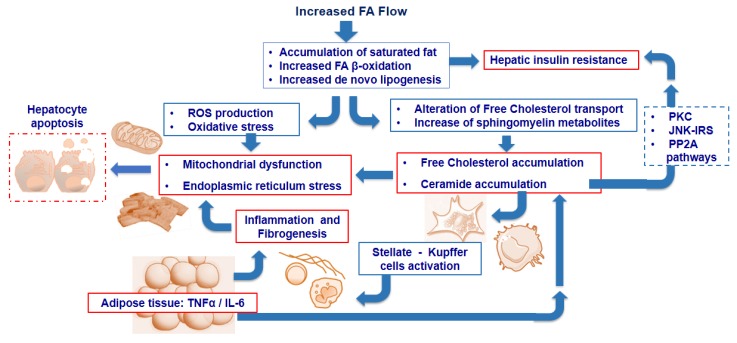
Excess fat accumulation promotes increased “*de novo lipogenesis*” (DNL) and fatty acid (FA) beta-oxidation. These mechanisms lead to reactive oxygen species (ROS) generation which induces oxidative mitochondrial damage and endoplasmic reticulum (ER) stress. In parallel, the accumulation of free (non-esterified) cholesterol and ceramides enhances both mitochondrial dysfunction and ER stress, and induces the activation of stellate cells as well as Kupffer cells, thus promoting inflammation and fibrosis. Furthermore, tumor necrosis factor alpha (TNFα) and interleukin-6 (IL-6) from adipose tissue enhance the inflammatory process and promotes ceramide accumulation, contributing to insulin resistance through different pathways. Ultimately, these events trigger the hepatocyte apoptotic pathway, leading to cell death. PKC: protein kinase; CJNK-IRS: c-Jun N-terminal kinase-insulin receptor substrate; PP2A: Protein Phosphatase 2A.

In addition to DAG, other lipid species as well as adipose tissue and intrahepatic inflammation and ER stress have been suggested to contribute to insulin resistance [23] (Figure 2).

Increases in hepatic and muscle ceramide content have been associated with insulin resistance in obese animals [24]. Ceramides may directly interact with protein kinase B and therefore have an effect on insulin signaling [25].

Adipose tissue and intrahepatic inflammation may also be involved in insulin resistance in subjects with NAFLD. Overexpression of tumor necrosis factor-alfa (TNF-α) in adipose tissue is a common correlate of TNF-α and insulin resistance in animal models [26]. In parallel, studies have demonstrated that obese individuals express 2.5-fold more TNF-α mRNA in fat tissue relative to lean controls [27]. A strong positive correlation has been observed between TNF-α mRNA expression levels in fat tissue and the extent of hyperinsulinemia, an indirect marker of insulin resistance [27]. On the other hand, in rodent models, high-fat diets and obesity have been shown to activate hepatic nuclear factor (NF)-κB [28,29], which cause hepatic inflammation, an increase in local and circulating interleukin (IL)-6, IL-1β, and TNF-α, with consequent hepatic and skeletal muscle insulin resistance [29]. In addition, administration of an antibody neutralizing IL-6 in mice on high-fat diets upregulates skeletal muscle glucose transport and modulates production of adipose tissue adipokines, resulting in improvement of hepatic insulin resistance and steatosis [30]. Taken together, these observations suggest that steatosis may activate intrahepatic inflammatory pathways that upregulate the production of proinflammatory cytokines leading to both hepatic and peripheral insulin resistance.

The ER stress response has recently been proposed to play a critical role in hepatic lipid accumulation, as well as in the pathogenesis of hepatic insulin resistance [31]. Activation of the ER-stress response can lead to hepatic steatosis by activating lipogenic pathways through its ability to stimulate several genes involved in lipid synthesis [31,32]. Moreover, this activation can cause hepatic insulin resistance through activation of the c-Jun N-terminal kinase (JNK) pathway, which inhibits insulin signaling through inactivation and/or degradation of IRS-1 [33]. It is known that ER stress is increased in the liver of obese individuals with NAFLD [34], and decreased in those with weight loss in parallel with an improvement in hepatic insulin sensitivity and the resolution of steatosis [35]. Yet, in both rodent models and human subjects [36,37], studies have demonstrated that treating obese mice as well as obese subjects with tauroursodeoxycholic acid, which acts as a chemical chaperone that reduces ER stress in liver and adipose tissue, results in improved insulin sensitivity in liver, muscle, and adipose tissue.

## 3. Lipid and Lipoprotein Metabolism in Nonalcoholic Fatty Liver Disease

Fat accumulation in the liver occurs when the rate of hepatic triglyceride synthesis (as a result of increased hepatic FA uptake and esterification into triglycerides as well as of “de novo lipogenesis” of triglycerides from carbohydrate and protein metabolism) exceeds the rate of hepatic triglyceride catabolism (which depends upon FA oxidation and export of triglycerides as very low density lipoproteins (VLDLs)).

### 3.1. Fatty Acid Uptake

Hepatic lipid uptake is a function of substrate delivery and transport into the hepatocyte. The major source of FAs for the liver is the systemic plasma FAs pool, mainly derived from the lipolysis of subcutaneous adipose tissue triglycerides, and, to a small extent, from lipolysis of triglycerides in circulating lipoproteins [38,39]. In contrast, only about 5% and 20% of portal vein FAs originate from visceral fat lipolysis in lean and obese subjects, respectively [40]. It has been shown that the rate at which FAs are released into the systemic circulation increases with increasing fat mass in both men and women [41].

Regional mobilization of circulating triglycerides has been demonstrated to be altered in obese patients with NAFLD. Lipoprotein lipase (LpL) hydrolyzes circulating triglycerides, followed by tissue uptake through FA transport proteins. LpL activity appears to be blunted, in response to insulin, in adipose tissue of obese patients. In contrast, NAFLD is associated with increased LpL as well as FA transport proteins [38,39]. Taken together, these data suggest that alterations in adipose tissue lipolytic activity, regional hepatic lipolysis of circulating triglycerides and FA transport proteins are involved in the pathogenesis of hepatic steatosis and ectopic fat accumulation.

### 3.2. Hepatic de Novo Lipogenesis

The contribution of hepatic de novo lipogenesis in healthy individuals is minor. In fact, in healthy subjects, hepatic de novo lipogenesis in the fasting and postprandial states has been reported to account for less than 5% and 10% of FAs incorporated into intra-hepatic triglycerides (IHTG) and VLDL-triglyceride, respectively [42]. In patients with NAFLD, the rate of hepatic de novo lipogenesis in the fasting state is also quite small and therefore is unlikely to be primarily responsible for the excessive liver fat accumulation. However, in these patients the rate increases in the postprandial state [42,43]. In particular, large increases in de novo lipogenesis following meal ingestion have been shown to precede excessive liver fat accumulation [44]. Importantly, specific dietary habits such as high carbohydrate meal and increased consumption of fructose, have been implicated in this increased rate and therefore in the development of NAFLD and its progression to NASH.

### 3.3. Fatty Acid Oxidation

The liver derives its source of energy mainly from oxidation of FAs. The oxidation of intra-hepatic FAs occurs primarily within mitochondria *beta*-oxidation and, to a much smaller extent, in peroxisomes and microsomes. *Beta*-oxidation requires FAs to be transported from the cytoplasm to the mitochondrial matrix, i.e., across the mitochondrial double membrane. This process needs the “activation” of FAs by co-enzyme A, which is accomplished by fatty acyl-CoA synthetase in the cytoplasm [45]. Then, carnitine palmitoyl transferase (CPT1) converts fatty acyl-CoA to fatty acyl carnitine in the outer mitochondrial membrane, which is eventually shuttled across the inner mitochondrial membrane by carnitine translocase [46]. Of great interest, targeting mitochondrial FAs oxidation through CPT1 in mouse models has been suggested as a strategy to treat obesity-related disorders including NAFLD [47]. Hepatic *beta*-oxidation/ketogenesis has been suggested to occur in patients with NAFLD [38,39,42,43]. Studies performed in rodent models have demonstrated that modulation (up- or down-regulation) of intra-hepatic oxidation of FAs by various stimuli influences IHTG content. Accordingly, genetic or experimentally-induced deficiency of hepatic enzymes involved in mitochondrial *beta*-oxidation may cause accumulation of IHTG [48], while an increase in the expression or activity of hepatic enzymes responsible of oxidation of FAs may decrease the accumulation of IHTG [49]. However, human studies have not led to consistent conclusions. In fact, while subjects with NAFLD have been reported to have hepatic mitochondrial structural and functional abnormalities (such as loss of mitochondrial cristae and paracrystalline inclusions [50,51], decreased mitochondrial respiratory chain activity [52], impaired ability to resynthesize ATP following a fructose challenge [53], and increased hepatic uncoupling protein 2 [54]), all of these abnormalities could affect the production of hepatic energy but not oxidation of FAs, possibly representing an adaptive uncoupling of oxidation of FAs and ATP production, which allows the liver to oxidize excessive fatty acid substrates, without generating unneeded ATP [51,52,53,54].

### 3.4. Very Low Density Lipoproteins Secretion

Hepatic secretion of triglycerides in the form of VLDL particles for delivery to peripheral tissues (including skeletal muscle, cardiac muscle and adipose tissue) is an important pathway for the mobilization of liver fat. The VLDL secretion rate appears to depend, not only on the availability of hepatic triglycerides, but also on the overall capacity for VLDL assembly. Hepatic VLDL assembly comprises two steps, both of which require the action of microsomal triglyceride transfer protein [55]. The first step involves the partial lipidation of a newly synthesized apolipoprotein (apo) B-100 molecule to form a small and dense VLDL precursor. In the second step, this small and dense precursor combines with a large triglyceride droplet to form a mature and triglyceride-rich VLDL, which is subsequently secreted into plasma [55,56,57]. Each VLDL particle contains one molecule of apoB100, which is required to export VLDL from the liver. The mechanisms responsible for the inadequate export of hepatic VLDL-triglyceride in patients with NAFLD are unknown. In such patients, the secretion rate of VLDL-triglyceride is much higher than that encountered in subjects without NAFLD [58], while VLDL particles (VLDL-apoB100) have been found to be either not different [58] or only slightly greater [59]. This suggests that VLDL particles produced by NAFLD patients may contain more triglycerides and may be larger than those produced by normal subjects. Animal studies have demonstrated that very large VLDL particles cannot be secreted from the liver because they exceed the diameter of the sinusoidal endothelial pores. Therefore, this inability can result in excessive IHTG accumulation, leading to hepatic steatosis [60]. In a very recent study, Fabbrini et al. [61] evaluated the physiologic mechanisms of weight gain-induced steatosis in 27 subjects. They found that weight gain increased the export of triglycerides from the liver by secreting a higher number of triglyceride-rich VLDL particles. However, this increase was not sufficient to fully compensate for the increased rate of IHTG production [61].

## 4. Role of Cholesterol in the Pathogenesis of Nonalcoholic Fatty Liver Disease/Nonalcoholic Steatohepatitis

Recent data suggest that disturbed hepatic cholesterol homeostasis and liver free cholesterol accumulation are important for the pathogenesis of NAFLD/NASH [62,63,64]. Hepatic free cholesterol accumulation in NAFLD results from alterations in intracellular cholesterol transport as well as from unbalanced cellular cholesterol homeostasis, characterized by the activation of cholesterol biosynthetic pathways, increased cholesterol de-esterification and attenuation of cholesterol export and bile acid synthesis pathways [65]. In this respect, ER stress triggers the release of transcription factors, such as sterol regulatory element binding protein-1c (SREBP-1c) and SREBP-2 in insulin-resistance, playing a relevant role in the synthesis of FAs and cholesterol, respectively [66,67,68,69].

Free cholesterol accumulation leads to liver injury through the activation of intracellular signaling pathways in Kupffer cells (KCs), Stellate cells (HSCs) and hepatocytes. The activation of KCs and HSCs promotes inflammation and fibrogenesis [70]. In addition, free cholesterol accumulation in the liver mitochondria induces mitochondrial dysfunction due to changes in membrane dynamics and direct interaction with proteins, which alters its activity. Increases in mitochondrial cholesterol disrupt membrane fluidity or dynamics and impair mitochondrial GSH transport [62]. This results in increased production of reactive oxygen species, and triggers the unfolded protein response in the ER, with consequent ER stress and apoptosis [71]. These events create a vicious circle contributing to the maintenance of steatosis and promoting ongoing hepatocyte death and liver damage, which in turn may lead to disease progression [72].

## 5. Role of Ceramides in Nonalcoholic Fatty Liver Disease

Ceramides are members of the sphingolipid family of lipids, and constitute an integral part of the structure of the cell membrane lipid bilayer [73]. However, ceramides also have cell signaling properties, and their accumulation in the liver, especially during periods of increased hepatic influx of free FAs, may contribute to insulin resistance [25]. Ceramides are important second messengers that interact with several pathways affecting insulin signaling, mitochondrial function, FA oxidation, and therefore promoting more inflammation, oxidative stress, inflammation, and cell death, all of which are linked to NAFLD [74]. Several risk factors for the progression of NAFLD, such as increased inflammatory cytokines (i.e., TNF-α and IL-6) along with oxidative stress, and decreased adiponectin have been associated with ceramide production in hepatocytes [75]. It is likely that the increase in inflammatory cytokines and oxidative stress is conducive to the generation of ceramides. Then, ceramides could further fuel the cellular damage caused by inflammation, promoting mitochondrial dysfunction, thus facilitating the development of NASH [76,77].

## 6. Medical Therapy

Medical treatments currently available in pediatric NAFLD are mainly aimed at (1) reducing body weight; (2) improving or preventing hepatic steatosis, inflammation and fibrosis; and (3) treating dyslipidemia [17]. For the purposes of this review, we will focus on pharmacological treatment of dyslipidemia.

### 6.1. Orlistat

It is the only Food and Drugs Administration (FDA)-approved drug labelled for weight loss in children above 12 years of age [78]. Orlistat inhibits pancreatic lipase to induce fat malabsorption. Common side effects are abdominal cramps, flatulence (due to unabsorbed fat in the fecal mass), interference with absorption of fat-soluble vitamins [17,79,80], and chronic kidney disease (due to secondary hyperoxaluria) [81].

### 6.2. Omega-3 Fatty Acids

Omega-3 are polyunsaturated (PU) FAs that regulate transcription factors related to hepatic lipid metabolism, leading to increased FA oxidation and down-regulation of pro-inflammatory genes [82]. Omega-3 FAs have been reported to lower circulating triglycerides, by decreasing the hepatic secretion of VLDL cholesterol or by increasing chylomicron metabolism. The effect of omega-3 FAs on triglycerides chiefly involves the suppression of hepatic VLDL apoB production and apoB pool size. Chan et al. [83] showed that fish oil supplementation comprising 45% eicosapentaenoic acid and 39% docosahexaenoic acid (DHA) lowered triglycerides (−18%) and VLDL apo B (−20%) and the hepatic secretion of VLDL apo B (−29%) compared with placebo. The percentage of conversions of VLDL apo B to intermediate-density lipoprotein (IDL) apo B, VLDL apoB to low density lipoprotein (LDL) apoB, and IDL apoB to LDL apoB also increased significantly. This effect determines a 35% reduction in triglyceride synthesis and an increase in FA mitochondrial oxidation. In particular, omega-3 FAs induce the aggregation of apoB after its exit from the ER, but before it leaves the Golgi. Upon exit from the Golgi, this aggregate material becomes extensively oxidized, and converted into large aggregates. The aggregates slowly degrade by an autophagic process [84]. In a study using cultured hepatocytes, the effects of incubation with palmitic acid, oleic acid, and DHA on ER stress and apoB-100 secretion were compared [85]. The investigators found that both oleic acid and palmitic acid decrease the secretion of apoB-100 by inducing ER stress; palmitic acid was more potent, both in terms of dose and time of exposure, most likely due to its ability to increase the synthesis of ceramide. In contrast, DHA which was the most potent of the three FAs in terms of inhibiting apoB-100 secretion via stimulation of autophagy, did not induce ER stress. Furthermore, it has been shown that DHA may interfere with apoCIII gene transcription, thereby potentially decreasing the negative effect of apoCIII on LpL activity [86].

A few studies have investigated the effects of n-3 long chain-PUFAs on pediatric NAFLD [87,88]. Nobili et al. reported a short-term (six months) [87] and long-term (up to 24 months) [88] effects of DHA, after 6, 12, 18, and 24 months of treatment with different concentrations (DHA 250 mg/day and 500 mg/day) combined with diet and exercise. In these studies, algae DHA supplementation improved liver steatosis and was able to reduce the levels of serum alanine aminotransferase and triglycerides. More recently, Nobili et al. found that treatment with DHA (250 mg/day) for 18 months improved histopathological parameters such as hepatocyte steatosis, ballooning, and NAFLD activity score but was ineffective on fibrosis [89]. Pacifico et al. investigated the effect of six months of DHA on liver fat in 58 children with biopsy-proven NAFLD [90]. Hepatic fat fraction as measured by magnetic resonance imaging decreased significantly in the group receiving DHA in comparison with that receiving placebo.

### 6.3. Ezetimibe

The FDA has recently approved ezetimibe use for treatment of hypercholesterolemia in children above 10 years. Ezetimibe selectively inhibits cholesterol absorption from intestine by binding to the brush border, and consistently lowers LDL-cholesterol [91]. Ezetimibe has also been shown to decrease hepatic lipid synthesis and favor lipid removal from the liver by preventing the degradation of microsomal triglyceride transfer protein, with a consequent inhibition of the development of NAFLD in animal models [92,93]. These results suggest that ezetimibe can increase lipoprotein assembly in the liver and perhaps in the intestine. Studies in adult patients with NAFLD have shown improvement in hepatic histology but with worsening or no effects on insulin sensitivity [94,95]. Promising results have been demonstrated when ezetimibe has been used in combination with statins [92]. No studies are available in children with NAFLD.

### 6.4. Statins

Statins are specific inhibitors of 3-hydroxy-3methylglutaryl coenzyme A reductase, the rate-limiting enzyme in cholesterol biosynthesis [96]. They also promote the synthesis of LDL-receptor (LDL-r) acting on the LDL-r gene, ultimately increasing the expression of membrane LDL-r [91]. In addition to their cholesterol-lowering effects, statins also possess pleiotropic properties that account for their anti-inflammatory, anti-proliferative, anti-thrombotic, anti-oxidative, anti-cancer, and immuno- modulatory actions in vitro and in vivo [97]. Statins can reduce liver triglycerides [98] and ameliorate severe hepatic steatosis [99]. The American Association for the Study of Liver Disease Guidelines recommends that statins can be used for treatment of dyslipidemia in patients with NAFLD and NASH [100]. Statins are now considered as a first line pharmacological intervention for pediatric patients with severe dyslipidemias [101]. However, no data are available in pediatric NAFLD.

### 6.5. Farnesoid X Receptor (FXR) Agonists

The farnesoid X receptor (FXR) is a bile acid-activated nuclear receptor. When bound to the FXR, lipophilic bile acids improve insulin sensitivity and decrease hepatic gluconeogenesis and circulating triglycerides [102]. These effects are mediated by decreased hepatic lipid synthesis and increased peripheral clearance of VLDL [103,104,105]. Thus, targeting FXR may offer new perspectives for the treatment of NAFLD. Support comes from studies investigating the effects of FXR activation by 6-ethyl-chenodeoxycholic acid, a potent activator of FXR, in rats with diabetes mellitus, obesity, insulin resistance, and liver steatosis [106]. FXR activation protected against body weight gain and liver and muscle fat deposition, and reversed insulin resistance. Activation of FXR reduced liver expression of genes involved in FA synthesis, lipogenesis and gluconeogenesis. Preliminary data in adult patients with NAFLD treated with 6-ethyl-chenodeoxycholic acid (obeticholic acid) have shown promising results [107], but its long-term benefits and safety need clarification. There are no data in pediatric populations.

## 7. Conclusions

NAFLD is one of the most prevalent chronic liver disorders in children and adolescents. The disease develops when the rate of hepatic triglyceride synthesis (as a result of increased hepatic fatty acid uptake and esterification into triglycerides as well as of “de novo lipogenesis” of triglycerides from carbohydrate and protein metabolism) exceeds the rate of hepatic triglyceride catabolism (which depends upon fatty acid oxidation and export of triglycerides as VLDLs). Genetic and physiological mechanisms regulating these processes become deregulated in the presence of nutritional oversupply, leading to development of NAFLD and hepatic insulin resistance. Hepatic insulin resistance is associated with increases in intra-hepatic triglyceride and DAG concentrations, with the latter being responsible for activation of PKC-epsilon and the subsequent inhibition of IRTK activity. Furthermore, in addition to DAG, other lipid species as well as adipose tissue and intrahepatic inflammation and ER stress have been suggested to contribute to hepatic insulin resistance. Therapies targeted to lipid and lipoprotein metabolism would be effective options for treating NAFLD and its cardiometabolic complications.

## References

[1] Angulo P. (2002). Nonalcoholic fatty liver disease. N. Engl. J. Med..

[2] Mencin A.A., Lavine J.E. (2011). Advances in pediatric nonalcoholic fatty liver disease. Pediatr. Clin. N. Am..

[3] Loomba R., Sirlin C.B., Schwimmer J.B., Lavine J.E. (2009). Advances in pediatric nonalcoholic fatty liver disease. Hepatology.

[4] Anderson E.L., Howe L.D., Jones H.E., Higgins J.P., Lawlor D.A., Fraser A. (2015). The Prevalence of Non-Alcoholic Fatty Liver Disease in Children and Adolescents: A Systematic Review and Meta-Analysis. PLoS ONE.

[5] Nobili V., Alisi A., Newton K.P., Schwimmer J.B. (2016). Comparison of the Phenotype and Approach to Pediatric vs Adult Patients With Nonalcoholic Fatty Liver Disease. Comparison of the Phenotype and Approach to Pediatric vs Adult Patients With Nonalcoholic Fatty Liver Disease. Gastroenterology.

[6] Rafiq N., Bai C., Fang Y., Srishord M., McCullough A., Gramlich T., Younossi Z.M. (2009). Long-Term Follow-Up of Patients With Nonalcoholic Fatty Liver. Clin. Gastroenterol. Hepatol..

[7] Byrne C.D. (2013). Ectopic fat, insulin resistance and non-alcoholic fatty liver disease. Proc. Nutr. Soc..

[8] Samuel V.T., Petersen K.F., Shulman G.I. (2010). Lipid-induced insulin resistance: Unraveling the mechanism. Lancet.

[9] Cali A.M., De Oliveira A.M., Kim H., Chen S., Reyes-Mugica M., Escalera S., Dziura J., Taksali S.E., Kursawe R., Shaw M. (2009). Glucose dysregulation and hepatic steatosis in obese adolescents: Is there a link?. Hepatology.

[10] D’Adamo E., Cali A.M., Weiss R., Santoro N., Pierpont B., Northrup V., Caprio S. (2010). Central role of fatty liver in the pathogenesis of insulin resistance in obese adolescents. Diabetes Care.

[11] Kotronen A., Yki-Järvinen H. (2008). Fatty liver: A novel component of the metabolic syndrome. Arterioscler. Thromb. Vasc. Biol..

[12] Schwimmer J.B., Pardee P.E., Lavine J.E., Blumkin A.K., Cook S. (2008). Cardiovascular risk factors and the metabolic syndrome in pediatric nonalcoholic fatty liver disease. Circulation.

[13] Pacifico L., Nobili V., Anania C., Verdecchia P., Chiesa C. (2011). Pediatric nonalcoholic fatty liver disease, metabolic syndrome and cardiovascular risk. World J. Gastroenterol..

[14] Targher G., Day C.P., Bonora E. (2010). Risk of cardiovascular disease in patients with nonalcoholic fatty liver disease. N. Engl. J. Med..

[15] Pacifico L., Anania C., Martino F., Cantisani V., Pascone R., Marcantonio A., Chiesa C. (2010). Functional and morphological vascular changes in pediatric nonalcoholic fatty liver disease. Hepatology.

[16] Pacifico L., Di Martino M., De Merulis A., Bezzi M., Osborn J.F., Catalano C., Chiesa C. (2014). Left ventricular dysfunction in obese children and adolescents with nonalcoholic fatty liver disease. Hepatology.

[17] Clemente M.G., Mandato C., Poeta M., Vajro P. (2016). Pediatric non-alcoholic fatty liver disease: Recent solutions, unresolved issues, and future research directions. World J. Gastroenterol..

[18] Buzzetti E., Pinzani M., Tsochatzis E.A. (2016). The multiple-hit pathogenesis of non-alcoholic fatty liver disease (NAFLD). Metabolism.

[19] Samuel V.T., Liu Z.X., Qu X., Elder B.D., Bilz S., Befroy D., Romanelli A.J., Shulman G.I. (2004). Mechanism of hepatic insulin resistance in non-alcoholic fatty liver disease. J. Biol. Chem..

[20] Samuel V.T., Liu Z.X., Wang A., Beddow S.A., Geisler J.G., Kahn M., Zhang X.M., Monia B.P., Bhanot S., Shulman G.I. (2007). Inhibition of protein kinase Cε prevents hepatic insulin resistance in nonalcoholic fatty liver disease. J. Clin. Invest..

[21] Kumashiro N., Erion D.M., Zhang D., Kahn M., Beddow S.A., Chu X., Still C.D., Gerhard G.S., Han X., Dziura J. (2011). Cellular mechanism of insulin resistance in nonalcoholic fatty liver disease. Proc. Natl. Acad. Sci. USA.

[22] Magkos F., Su X., Bradley D., Fabbrini E., Conte C., Eagon J.C., Varela J.E., Brunt E.M., Patterson B.W., Klein S. (2012). Intrahepatic diacylglycerol content is associated with hepatic insulin resistance in obese subjects. Gastroenterology.

[23] Birkenfeld A.L., Shulman G.I. (2014). Nonalcoholic Fatty Liver Disease, Hepatic Insulin Resistance, and Type 2 Diabetes. Hepatology.

[24] Turinsky J., Bayly B.P., O’Sullivan D.M. (1990). 1,2-Diacylglycerol and ceramide levels in rat skeletal muscle and liver in vivo. Studies with insulin, exercise, muscle denervation, and vasopressin. J. Biol. Chem..

[25] Chavez J.A., Summers S.A. (2012). A ceramide-centric view of insulin resistance. Cell Metab..

[26] Hotamisligil G.S., Shargill N.S., Spiegelman B.M. (1993). Adipose expression of tumor necrosis factor-alpha: Direct role in obesity-linked insulin resistance. Science.

[27] Hotamisligil G.S., Arner P., Caro J.F., Atkinson R.L., Spiegelman B.M. (1995). Increased adipose tissue expression of tumor necrosis factor-alpha in human obesity and insulin resistance. J. Clin. Investig..

[28] Muoio D.M., Newgard C.B. (2008). Mechanisms of disease: Molecular and metabolic mechanisms of insulin resistance and beta-cell failure in type 2 diabetes. Nat. Rev. Mol. Cell Biol..

[29] Cai D., Yuan M., Frantz D.F., Melendez P.A., Hansen L., Lee J., Shoelson S.E. (2005). Local and systemic insulin resistance resulting from hepatic activation of IKK-β and NF-κB. Nat. Med..

[30] Yamaguchi K., Nishimura T., Ishiba H., Seko Y., Okajima A., Fujii H., Tochiki N., Umemura A., Moriguchi M., Sumida Y. (2015). Blockade of interleukin 6 signalling ameliorates systemic insulin resistance through upregulation of glucose uptake in skeletal muscle and improves hepatic steatosis in high-fat diet fed mice. Liver Int..

[31] Zhang X.Q., Xu C.F., Yu C.H., Chen W.X., Li Y.M. (2014). Role of endoplasmic reticulum stress in the pathogenesis of nonalcoholic fatty liver disease. World J. Gastroenterol..

[32] Wang D., Wei Y., Pagliassotti M.J. (2006). Saturated fatty acids promote endoplasmic reticulum stress and liver injury in rats with hepatic steatosis. Endocrinology.

[33] Ozcan U., Cao Q., Yilmaz E., Lee A.H., Iwakoshi N.N., Ozdelen E., Tuncman G., Gorgun C., Glimcher L.H., Hotamisligil G.S. (2004). Endoplasmic reticulum stress links obesity, insulin action, and type 2 diabetes. Science.

[34] Puri P., Mirshahi F., Cheung O., Natarajan R., Maher J.W., Kellum J.M., Sanyal A.J. (2008). Activation and dysregulation of the unfolded protein response in nonalcoholic fatty liver disease. Gastroenterology.

[35] Gregor M.F., Yang L., Fabbrini E., Mohammed B.S., Eagon J.C., Hotamisligil G.S., Klein S. (2009). Endoplasmic reticulum stress is reduced in tissues of obese subjects after weight loss. Diabetes.

[36] Ozcan U., Yilmaz E., Ozcan L., Furuhashi M., Vaillancourt E., Smith R.O., Görgün C.Z., Hotamisligil G.S. (2006). Chemical chaperones reduce ER stress and restore glucose homeostasis in a mouse model of type 2 diabetes. Science.

[37] Kars M., Yang L., Gregor M.F., Mohammed B.S., Pietka T.A., Finck B.N., Patterson B.W., Horton J.D., Mittendorfer B., Hotamisligil G.S. (2010). Tauroursodeoxycholic Acid may improve liver and muscle but not adipose tissue insulin sensitivity in obese men and women. Diabetes.

[38] Fabbrini E., Magkos F. (2015). Hepatic steatosis as a marker of metabolic dysfunction. Nutrients.

[39] Fabbrini E., Sullivan S., Klein S. (2010). Obesity and nonalcoholic fatty liver disease: Biochemical, metabolic, and clinical implications. Hepatology.

[40] Nielsen S., Guo Z., Johnson C.M., Hensrud D.D., Jensen M.D. (2004). Splanchnic lipolysis in human obesity. J. Clin. Investig..

[41] Mittendorfer B., Magkos F., Fabbrini E., Mohammed B.S., Klein S. (2009). Relationship between body fat mass and free fatty acid kinetics in men and women. Obesity.

[42] Diraison F., Moulin P., Beylot M. (2003). Contribution of hepatic de novo lipogenesis and reesterification of plasma non esterified fatty acids to plasma triglyceride synthesis during non-alcoholic fatty liver disease. Diabetes Metab..

[43] Donnelly K.L., Smith C.I., Schwarzenberg S.J., Jessurun J., Boldt M.D., Parks E.J. (2005). Sources of fatty acids stored in liver and secreted via lipoproteins in patients with nonalcoholic fatty liver disease. J. Clin. Investig..

[44] Petersen K.F., Dufour S., Savage D.B., Bilz S., Salomon G., Yonemitsu S., Cline G.W., Befroy D., Zemany L., Kahn B.B. (2007). The role of skeletal muscle insulin resistance in the pathogenesis of the metabolic syndrome. Proc. Natl. Acad. Sci. USA.

[45] Wanders R.J., Komen J., Kemp S. (2011). Fatty acid omega-oxidation as a rescue pathway for fatty acid oxidation disorders in humans. FEBS J..

[46] McGarry J.D., Woeltje K.F., Kuwajima M., Foster D.W. (1989). Regulation of ketogenesis and the renaissance of carnitine palmitoyltransferase. Diabetes Metab. Rev..

[47] Serra D., Mera P., Malandrino M.I., Mir J.F., Herrero L. (2013). Mitochondrial fatty acid oxidation in obesity. Antioxid. Redox Signal..

[48] Zhang D., Liu Z.X., Choi C.S., Tian L., Kibbey R., Dong J., Cline G.W., Wood P.A., Shulman G.I. (2007). Mitochondrial dysfunction due to long-chain Acyl-CoA dehydrogenase deficiency causes hepatic steatosis and hepatic insulin resistance. Proc. Natl. Acad. Sci. USA.

[49] Da Silva R.P., Kelly K.B., Leonard K.A., Jacobs R.L. (2014). Creatine reduces hepatic TG accumulation in hepatocytes by stimulating fatty acid oxidation. Biochim. Biophys. Acta.

[50] Caldwell S.H., Swerdlow R.H., Khan E.M., Iezzoni J.C., Hespenheide E.E., Parks J.K., Parker W.D. (1999). Mitochondrial abnormalities in non-alcoholic steatohepatitis. J. Hepatol..

[51] Sanyal A.J., Campbell-Sargent C., Mirshahi F., Rizzo W.B., Contos M.J., Sterling R.K., Luketic V.A., Shiffman M.L., Clore J.N. (2001). Nonalcoholic steatohepatitis: Association of insulin resistance and mitochondrial abnormalities. Gastroenterology.

[52] Perez-Carreras M., Del Hoyo P., Martin M.A., Rubio J.C., Martin A., Castellano G., Colina F., Arenas J., Solis-Herruzo J.A. (2003). Defective hepatic mitochondrial respiratory chain in patients with nonalcoholic steatohepatitis. Hepatology.

[53] Cortez-Pinto H., Chatham J., Chacko V.P., Arnold C., Rashid A., Diehl A.M. (1999). Alterations in liver ATP homeostasis in human nonalcoholic steatohepatitis: A pilot study. JAMA.

[54] Kohjima M., Enjoji M., Higuchi N., Kato M., Kotoh K., Yoshimoto T., Fujino T., Yada M., Yada R., Harada N. (2007). Re-evaluation of fatty acid metabolism-related gene expression in nonalcoholic fatty liver disease. Int. J. Mol. Med..

[55] Shelness G.S., Sellers J.A. (2001). Very-low-density lipoprotein assembly and secretion. Curr. Opin. Lipidol..

[56] Elovson J., Chatterton J.E., Bell G.T., Schumaker V.N., Reuben M.A., Puppione D.L., Reeve J.R., Young N.L. (1988). Plasma very low density lipoproteins contain a single molecule of apolipoprotein B. J. Lipid Res..

[57] Packard C.J. (1999). Understanding coronary heart disease as a consequence of defective regulation of apolipoprotein B metabolism. Curr. Opin. Lipidol..

[58] Fabbrini E., Mohammed B.S., Magkos F., Korenblat K.M., Patterson B.W., Klein S. (2008). Alterations in adipose tissue and hepatic lipid kinetics in obese men and women with nonalcoholic fatty liver disease. Gastroenterology.

[59] Chan D.C., Watts G.F., Gan S., Wong A.T., Ooi E.M., Barrett P.H. (2010). Nonalcoholic fatty liver disease as the transducer of hepatic oversecretion of very-low-density lipoprotein-apolipoprotein B-100 in obesity. Arterioscler. Thromb. Vasc. Biol..

[60] Horton J.D., Shimano H., Hamilton R.L., Brown M.S., Goldstein J.L. (1999). Disruption of LDL receptor gene in transgenic SREBP-1a mice unmasks hyperlipidemia resulting from production of lipid-rich VLDL. J. Clin. Investig..

[61] Fabbrini E., Tiemann Luecking C., Love-Gregory L., Okunade A.L., Yoshino M., Fraterrigo G., Patterson B.W., Klein S. (2016). Physiological Mechanisms of Weight Gain-Induced Steatosis in People With Obesity. Gastroenterology.

[62] Ioannou G.N. (2015). The role of cholesterol in the pathogenesis of NASH. Trends Endocrinol. Metab..

[63] Van Rooyen D.M., Larter C.Z., Haigh V.G., Yeh M.M., Iannou G., Kuver R., Lee S.P., Tech N.C., Farrell G.C. (2011). Hepatic free cholesterol accumulates in obese, diabetic mice and causes nonalcoholic steatohepatitis. Gastroenterology.

[64] Caballero F., Fernández A., De Lacy A.M., Fernández-Checa J.C., Caballería J., García-Ruiz C. (2009). Enhanced free cholesterol, SREBP-2 and StAR expression in human NASH. J. Hepatol..

[65] Nuño-Lámbarri N., Domínguez-Pérez M., Baulies-Domenech A., Monte M.J., Marin J.J., Rosales-Cruz P., Souza V., Miranda R.U., Bucio L., Montalvo-Jave E.E. (2016). Liver Cholesterol Overload Aggravates Obstructive Cholestasis by Inducing Oxidative Stress and Premature Death in Mice. Oxid. Med. Cell. Longev..

[66] Brown M.S., Goldstein J.L. (1997). The SREBP pathway: Regulation of cholesterol metabolism by proteolysis of a membrane-bound transcription factor. Cell.

[67] Browning J.D., Horton J.D. (2004). Molecular mediators of hepatic steatosis and liver injury. J. Clin. Investig..

[68] Horton J.D., Goldstein J.L., Brown M.S. (2002). SREBPs: Activators of the complete program of cholesterol and fatty acid synthesis in the liver. J. Clin. Investig..

[69] Flamment M., Hajduch E., Ferré P., Foufelle F. (2012). New insights into ER stress-induced insulin resistance. Trends Endocrinol. Metab..

[70] Tomita K., Teratani T., Suzuki T., Shimizu M., Sato H., Narimatsu K., Okada Y., Kurihara C., Irie R., Yokoyama H. (2014). Free cholesterol accumulation in hepatic stellate cells: mechanism of liver fibrosis aggravation in nonalcoholic steatohepatitis in mice. Hepatology.

[71] Mari M., Caballero F., Colell A., Morales A., Caballeria J., Fernandez A., Enrich C., Fernandez-Checa J.C., Garcia-Ruiz C. (2006). Mitochondrial free cholesterol loading sensitizes to TNF- and Fas-mediated steatohepatitis. Cell Metab..

[72] Wouters K., van Bilsen M., van Gorp P.J., Bieghs V., Lutjohann D., Kerksiek A., Staels B., Hofker M.H., Shiri-Sverdlov R. (2010). Intrahepatic cholesterol influences progression, inhibition and reversal of non-alcoholic steatohepatitis in hyperlipidemic mice. FEBS Lett..

[73] Gault C.R., Obeid L.M., Hannun Y.A. (2010). An overview of sphyngolipid metabolism: from synthesis to breakdown. Adv. Exp. Med. Biol..

[74] Reali F., Morine M.J., Kahramanoğulları O., Raichur S., Schneider H.C., Crowther D., Priami C. (2017). Mechanistic interplay between ceramide and insulin resistance. Sci. Rep..

[75] Correnti J.M., Juskeviciute E., Swarup A., Hoek J.B. (2014). Pharmacological ceramide reduction alleviates alcohol-induced steatosis and hepatomegaly in adiponectin knockout mice. Am. J. Physiol. Gastrointest. Liver Physiol..

[76] Fucho R., Casals N., Serra D., Herrero L. (2017). Ceramides and mitochondrial fatty acid oxidation in obesity. FASEB J..

[77] Pagadala M., Kasumov T., McCullough A.J., Zein N.N., Kirwan J.P. (2012). Role of ceramides in nonalcoholic fatty liver disease. Trends Endocrinol. Metab..

[78] Barlow S.E., Expert Committee (2007). Expert committee recommendations regarding the prevention, assessment, and treatment of child and adolescent overweight and obesity: summary report. Pediatrics.

[79] Huang J.S., Barlow S.E., Quiros-Tejeira R.E., Scheimann A., Skelton J., Suskind D., Tsai P., Uko V., Warolin J.P., Xanthakos S.A. (2013). Childhood obesity for pediatric gastroenterologists. J. Pediatr. Gastroenterol. Nutr..

[80] Sjöström L., Narbro K., Sjöström C.D., Karason K., Larsson B., Wedel H., Lystig T., Sullivan M., Bouchard C., Carlsson B. (2007). Effects of bariatric surgery on mortality in Swedish obese subjects. N. Engl. J. Med..

[81] Kwan T.K., Chadban S.J., McKenzie P.R., Saunders J.R. (2013). Acute oxalate nephropathy secondary to orlistat-induced enteric hyperoxaluria. Nephrology.

[82] Pacifico L., Giansanti S., Gallozzi A., Chiesa C. (2014). Long chain omega-3 polyunsaturated fatty acids in pediatric metabolic syndrome. Mini Rev. Med. Chem..

[83] Chan D.C., Watts G.F., Mori T.A., Barrett P.H., Redgrave T.G., Beilin L.J. (2003). Randomized controlled trial of the effect of n-3 fatty acid supplementation on the metabolism of apolipoprotein B-100 and chylomicron remnants in men with visceral obesity. Am. J. Clin. Nutr..

[84] Pan M., Maitin V., Parathath S., Andreo U., Lin S.X., St. Germain C., Yao Z., Maxfield F.R., Williams K.J., Fisher E.A. (2008). Presecretory oxidation, aggregation, and autophagic destruction of apoprotein-B: a pathway for late-stage quality control. Proc. Natl. Acad. Sci. USA.

[85] Caviglia J.M., Gayet C., Ota T., Hernandez-Ono A., Conlon D.M., Jiang H., Fisher E.A., Ginsberg H.N. (2011). Different fatty acids inhibit apoB100 secretion by different pathways: unique roles for ER stress, ceramide, and autophagy. J. Lipid Res..

[86] Olivieri O., Martinelli N., Sandri M., Bassi A., Guarini P., Trabetti E., Pizzolo F., Girelli D., Friso S., Pignatti P.F. (2005). Apolipoprotein C-III, n-3 polyunsaturated fatty acid, and “insulin-resistant” T-455C *APOC3* gene polymorphism in heart disease patients: example of gene-diet interaction. Clin. Chem..

[87] Nobili V., Bedogni G., Alisi A., Pietrobattista A., Risé P., Galli C., Agostoni C. (2011). Docosahexaenoic acid supplementation decreases liver fat content in children with non-alcoholic fatty liver disease: Double-blind randomised controlled clinical trial. Arch. Dis. Child..

[88] Nobili V., Alisi A., Della Corte C., Risé P., Galli C., Agostoni C., Bedogni G. (2013). Docosahexaenoic acid for the treatment of fatty liver: Randomised controlled trial in children. Nutr. Metab. Cardiovasc. Dis..

[89] Nobili V., Carpino G., Alisi A., De Vito R., Franchitto A., Alpini G., Onori P., Gaudio E. (2014). Role of docosahexaenoic acid treatment in improving liver histology in pediatric nonalcoholic fatty liver disease. PLoS ONE.

[90] Pacifico L., Bonci E., Di Martino M., Versacci P., Andreoli G., Silvestri L.M., Chiesa C. (2015). A double-blind, placebo-controlled randomized trial to evaluate the efficacy of docosahexaenoic acid supplementation on hepatic fat and associated cardiovascular risk factors in overweight children with nonalcoholic fatty liver disease. Nutr. Metab. Cardiovasc. Dis..

[91] Chang Y., Robidoux J. (2017). Dyslipidemia management update. Curr. Opin. Pharmacol..

[92] Averna M. (2015). The effect of ezetimibe on NAFLD. Atheroscler. Suppl..

[93] Wang X., Sugimoto K., Fujisawa T., Shindo N., Minato S., Kamada Y., Hamano M., Ohishi M., Ikegami H., Rakugi H. (2014). Novel effect of ezetimibe to inhibit the development of non-alcoholic fatty liver disease in Fatty Liver Shionogi mouse. Hepatol. Res..

[94] Husain N.E., Hassan A.T., Elmadhoun W.M., Ahmed M.H. (2015). Evaluating the safety of Liptruzet (ezetimibe and atorvastatin): What are the potential benefits beyond low-density lipoprotein cholesterol-lowering effect?. Expert Opin. Drug Saf..

[95] Takeshita Y., Takamura T., Honda M., Kita Y., Zen Y., Kato K., Misu H., Ota T., Nakamura M., Yamada K. (2014). The effects of ezetimibe on non-alcoholic fatty liver disease and glucose metabolism: A randomised controlled trial. Diabetologia.

[96] Baigent C., Keech A., Kearney P.M., Blackwell L., Buck G., Pollicino C., Kirby A., Sourjina T., Peto R., Collins R. (2005). Efficacy and safety of cholesterol-lowering treatment: Prospective meta-analysis of data from 90,056 participants in 14 randomised trials of statins. Lancet.

[97] Chong L.W., Hsu Y.C., Lee T.F., Lin Y., Chiu Y.T., Yang K.C., Wu J.C., Huang Y.T. (2015). Fluvastatin attenuates hepatic steatosis-induced fibrogenesis in rats through inhibiting paracrine effect of hepatocyte on hepatic stellate cells. BMC Gastroenterol..

[98] Roglans N., Sanguino E., Peris C., Alegret M., Vazquez M., Adzet T., Díaz C., Hernández G., Laguna J.C., Sánchez R.M. (2002). Atorvastatin treatment induced peroxisome proliferator-activated receptor α expression and decreased plasma nonesterified fatty acids and liver triglyceride in fructose-fed rats. J. Pharmacol. Exp. Ther..

[99] Egawa T., Toda K., Nemoto Y., Ono M., Akisaw N., Saibara T., Hayashi Y., Hiroi M., Enzan H., Onishi S. (2003). Pitavastatin ameliorates severe hepatic steatosis in aromatase-deficient (Ar−/−) mice. Lipids.

[100] Chalasani N., Younossi Z., Lavine J.E., Diehl A.M., Brunt E.M., Cusi K., Charlton M., Sanyal A.J. (2012). The diagnosis and management of non-alcoholic fatty liver disease: Practice Guideline by the American Association for the Study of Liver Diseases, American College of Gastroenterology, and the American Gastroenterological Association. Hepatology.

[101] De Ferranti S.D., Rodday A.M., Parsons S.K., Cull W.L., O’Connor K.G., Daniels S.R., Leslie L.K. (2017). Cholesterol Screening and Treatment Practices and Preferences: A Survey of United States Pediatricians. J. Pediatr..

[102] Porez G., Prawitt J., Gross B., Staels B. (2012). Bile acid receptors as targets for the treatment of dyslipidemia and cardiovascular disease. J. Lipid Res..

[103] Modica S., Gadaleta R.M., Moschetta A. (2010). Deciphering the nuclear bile acid receptor FXR paradigm. Nucl. Recept. Signal..

[104] Calkin A.C., Tontonoz P. (2012). Transcriptional integration of metabolism by the nuclear sterol-activated receptors LXR and FXR. Nat. Rev. Mol. Cell Biol..

[105] De Aguiar Vallim T.Q., Tarling E.J., Edwards P.A. (2013). Pleiotropic roles of bile acids in metabolism. Cell Metab..

[106] Cipriani S., Mencarelli A., Palladino G., Fiorucci S. (2010). FXR activation reverses insulin resistance and lipid abnormalities and protects against liver steatosis in Zucker (*fa*/*fa*) obese rats. J. Lipid Res..

[107] Neuschwander-Tetri B.A., Loomba R., Sanyal A.J., Lavine J.E., Van Natta M.L., Abdelmalek M.F., Chalasani N., Dasarathy S., Diehl A.M., Hameed B. (2015). Farnesoid X nuclear receptor ligand obeticholic acid for non-cirrhotic, non-alcoholic steatohepatitis (FLINT): A multicentre, randomised, placebo-controlled trial. Lancet.

